# Biodistribution and radiodosimetry of a novel myocardial perfusion tracer ^123^I-CMICE-013 in healthy rats

**DOI:** 10.1186/2191-219X-4-16

**Published:** 2014-03-13

**Authors:** Yin Duan, Julia Lockwood, Lihui Wei, Chad Hunter, Karen Soueidan, Corinne Bensimon, Pasan Fernando, R Glenn Wells, Terrence D Ruddy

**Affiliations:** 1 Nordion Inc, 447 March Road, Ottawa, ON K2K 1X8, Canada; 2 Division of Cardiology, Faculty of Medicine, University of Ottawa Heart Institute, 40 Ruskin Street, Ottawa, ON K1Y 4 W7, Canada; 3 Canadian Molecular Imaging Center of Excellence (CMICE), University of Ottawa Heart Institute, 40 Ruskin Street, UOHI-H5228, Ottawa, ON K1Y 4 W7, Canada; 4 Department of Cellular and Molecular Medicine, Faculty of Medicine, University of Ottawa, Ottawa, ON K1H 8 M5, Canada

**Keywords:** ^123^I-CMICE-013, Myocardial perfusion imaging, Biodistribution, Radiodosimetry, Quantitative SPECT imaging

## Abstract

**Background:**

^123^I-CMICE-013 is a novel radiotracer previously reported to have promising characteristics for single-photon emission computed tomography (SPECT) myocardial perfusion imaging. We evaluated the biokinetics and radiodosimetry of this rotenone-like ^123^I-labeled tracer in a microSPECT imaging-based study.

**Methods:**

37 to 111 MBq of ^123^I-CMICE-013 was synthesized and administered intravenously to 14 healthy rats. Images were acquired with a microSPECT/CT camera at various time intervals and reconstructed to allow activity quantification in the tissues of interest. Radiation dosage resulted from the injection of ^123^I-CMICE-013 was estimated base on the biodistribution data. Tissue uptake values from image analysis were verified by gamma-counting dissected organs *ex vivo*.

**Results:**

The heart/stomach and heart/intestine uptake ratios peaked shortly after the injection of ^123^I-CMICE-013, meanwhile the heart/liver ratio reached 2 as early as at 23 min post-injection. Little activity was observed in the lung and overnight clearance was significant in most of the measured tissues. The radiation dosimetry analysis based on the time-activity curves provided an estimate of the effective human dose of 6.99E-03 mSv/MBq using ICRP 60 and 7.15E-03 mSv/MBq using ICRP 103, which is comparable to the popular myocardium perfusion imaging (MPI) agents such as ^99m^Tc-tetrofosmin and ^99m^Tc-sestamibi, as well as other ^123^I-based radiotracers.

**Conclusions:**

^123^I-CMICE-013 demonstrated desirable characteristics in its biokinetic and radiodosimetric profiles, supporting its potential application as a novel myocardial perfusion imaging agent.

## Background

The assessment of myocardial perfusion is an important component of the evaluation of patients with coronary artery disease (CAD). Compared to other non-invasive imaging modalities such as positron emission tomography, and cardiac magnetic resonance imaging, single-photon emission computed tomography (SPECT) remains the most commonly used modality for myocardial perfusion imaging (MPI) due to its cost effectiveness, wide availability of SPECT cameras and its well validated methodology.

MPI using ^201^Tl or ^99m^Tc-based agents (e.g. ^99m^Tc-sestamibi and ^99m^Tc-tetrofosmin) has been the dominant SPECT diagnostic method for identifying and characterizing CAD. However, the relatively low first-pass myocardial extraction of these ^99m^Tc tracers at high flow rates limits the contrast obtained between normal and abnormal tissues [1,2]. And their proximal gastrointestinal activity uptake can sometimes create unwanted scattering to the myocardium [1-4]. Usually considered as an alternative, ^201^Tl also has quite a few undesirable characteristics: the low energy of the primary emission peak at 80 keV results in increased attenuation of the signal [2], the long physical half-life of 73 h limits the injected dose and leads to both noisier images and a high radiation exposure (2.2E-01 mSv/MBq according to ICRP 80 [5]), and its continuous redistribution requires acquisition immediately following injection for accurate stress images [1,2].

Our group previously reported the radiolabeling and purification of an iodinated rotenone molecule ^123^I-CMICE-013, a radioiodinated derivative of rotenone [6]. This new radiotracer is a potential alternative to the conventionally used MPI tracers such as ^201^Tl, ^99m^Tc-sestamibi or ^99m^Tc-tetrofosmin. Preliminary experiments in healthy rats have demonstrated the clear visualization of the myocardium and limited background activity using microSPECT/CT imaging. Imaging with ^123^I-CMICE-013 of chronic occlusion and ischemia/reperfusion rat models demonstrated excellent images of myocardial defects, which correlated well with *ex vivo* staining with triphenyltetrazolium chloride (TTC) [6].

In the current study, we acquired microSPECT/CT images at various time points to better understand the *in vivo* kinetic profile of ^123^I-CMICE-013 in healthy rats and quantified the activity in various organs. Time-activity curves were constructed to evaluate the uptake of tracers in the myocardium and other organs. We compared the data from imaging-based analysis to those acquired through tissue dissection and gamma counter measurement, and verified the accuracy of the image-based quantification approach. Radiation dosimetry is an important metric for evaluating the safety of a novel radioactive imaging agent. The internal radiation exposure of ^123^I-CMICE-013 was assessed using the resident activities data from the imaging biodistribution.

## Methods

### Materials

Analytical grade chemicals and solvents were used in this study without further purification. Rotenone was purchased from Sigma Aldrich (St. Louis, MO, USA). Iodine-123 isotope was provided by Nordion Inc (Ottawa, ON, Canada). A preparative high-performance liquid chromatography (HPLC) system was assembled using Dionex GP50 gradient pump (Sunnyvale, CA, USA), Waters Fraction Collector III (Milford, MA, USA), and Rheodyne 7725i six-port sample injection valve. Analytical HPLC was performed on a Waters system (Milford, MA, USA) with a 1525 Pump, 2998 Photodiode Array Detector, and 717+ Autosampler. A PerkinElmer 150TR Flow Scintillation Analyzer (Waltham, MA, USA) was connected to the analytical HPLC system and utilized as a radio-signal detecting module. A Capintec CRC-25R Dose calibrator (Ramsey, NJ, USA) was used to measure the radioactivity of ^123^I-CMICE-013 injections.

### Radiosynthesis of ^123^I-CMICE-013

The radiosynthesis of ^123^I-CMICE-013 follows the method described in our previous publication [6]. Briefly, 463 MBq (12.5 mCi) of Iodine-123 (in the form of Na^123^I in 0.1 M NaOH) was added into a 1.5-mL microtest tube. Then, 170 μL of rotenone (2.5 mg/mL, dissolved in trifluoroacetic acid) and 30 μL iodogen (0.75 mg/mL, dissolved in trifluoroacetic acid) were added sequentially, with the resulted molar ratio of rotenone versus iodogen at 21:1. The reaction was carried out at 60°C with mixing at 600 rpm for 45 min. After brief cooling, reaction mixture was injected into the preparative HPLC system consisting of Luna C18 (2) reversed phase column (Phenomenex, Torrance, CA, USA). Mobile phase ethanol/water (50%/50%, *v*/*v*) was pumped into the system at the flow rate of 0.7 mL/min and fraction collecting (0.7 mL/fraction) began right after the injection. The identity/radiochemical purity (RCP) of the collected product was first examined on analytical HPLC and then underwent re-formulation. The final product of ^123^I-CMICE-013 was re-constituted in a 5% EtOH (*v*/*v*) 10 mM NaOAc at pH 6.5. The radiochemical purity of the final product was ≥95%, pH was between 3 and 7, and the alcohol percentage was between 3% and 7%.

### ^123^I-CMICE-013 microSPECT/CT imaging

All animal experiments were conducted in compliance with the guidelines of the Canadian Council on Animal Care (CCAC) and with approval from the Animal Care Committee (ACC) at the University of Ottawa. For the study, a total of 14 Sprague–Dawley rats (Charles River Laboratories, Wilmington, MA, USA) weighing 251 ± 16 g were divided into two groups. Animals in group A (*n* = 6) were repeatedly scanned for 15-min acquisitions at 20-min intervals from immediately to 2 h after injection of ^123^I-CMICE-013. Rats in group B (*n* = 8) were first injected with the tracer, then allowed to recover from anesthesia before returning to regular housing, and imaged using two 1-h acquisitions at 24 h post-injection. The amount of injected tracer activity was between 37 and 111 MBq (1 to 3 mCi). All rats were sedated under light anesthesia with 1% to 2% isoflurane delivered through a nose cone during tracer injection as well as imaging.

A NanoSPECT/CT scanner (Bioscan, Washington, DC, USA) was used in the study. The scanner has 4 NaI detectors, each with a 9 × 2.5-mm-diameter multi-pinhole collimator. During imaging, the rats were placed in the supine position and their temperature maintained throughout the imaging session with a Minerva heated scanner bed (Bioscan, Washington, DC, USA). Projection data were acquired with a spiral scan covering the full body of the rat. A single 10-min whole-body CT scan was acquired prior to the SPECT acquisition for localization of tracer uptake. A calibrated source of ^123^I was placed within the SPECT field of view in order to facilitate image quantification. The animals' respiration and heart rate were monitored during the scanning process with a Model 1025 T Small Animal Monitoring System (BioScan, Washington DC, USA).

### Imaging biodistribution

Images were reconstructed using HiSPECT software provided by the camera manufacturer (BioScan, Washington, DC, USA). The reconstruction algorithm was ordered subset expectation maximization (OSEM) using a progressively small number of subsets: eight iterations of six subsets (8 × 6), followed by 8 × 4 and 8 × 1. The reconstructed voxel size was 0.6 × 0.6 × 0.6 mm. The reconstruction included collimator modeling but no correction for attenuation or scatter. Images were post-reconstruction filtered using a 3D Gaussian filter with a full width at half maximum of 0.63 mm. Images acquired at 3, 23, 43, 62, 82, and 102 min and 24 h after the radiotracer injection were analyzed with MATLAB (MathWorks, Boston, MA, USA). For each rat, the set of acquired images were summed together. Manual volumes of interest (VOI) were drawn around all areas of focal uptake in the summed image. The VOI was restricted to the region above soft-tissue background and applied separately to the images from each time point. The total counts in each VOI at each time point were decay-corrected and recorded. A VOI was designated around the ^123^I calibration source and the average decay-corrected counts in the source were used to convert the measured counts to activity. The activity in each VOI was associated with organs with the assistance of the CT scan. The percentage injected dose per organ (%ID/organ) at a certain time point were calculated by dividing the activity at the VOI region by the injection dose value as measured on the dose calibrator.

### Tissue biodistribution

Following image acquisition, animals were euthanized: group A at 2 h and group B at 24 h after tracer injection. Their heart, liver, kidney, muscle, femur, spleen, blood, brain, intestine, lung, stomach, urine/bladder, ovaries/testes, and thyroid were extracted, weighed, and analyzed for total gamma counts using a PerkinElmer Wizard3 2480 Automatic Gamma Counter (Waltham, MA, USA). The tissue uptake is represented as the %ID/organ.

### Dosimetry estimation

From the imaging biodistribution study, the %ID/organ values as a function of time were averaged over rats and integrated to calculate the residence times of the tracer in each organ. The rat residence times were converted into human residence times by multiplying the rat residence time by the mass factors as shown in the following equation [7]:

$${\left(\frac{\%\mathrm{ID}}{\text{organ}}\right)}_{\text{human}}=\left[{\left(\frac{\%\mathrm{ID}}{\text{organ}}\right)}_{\text{rat}}\times {\left(\frac{m_{\mathrm{TB}}}{m_{\text{organ}}}\right)}_{\text{rat}}\times {\left(\frac{m_{\text{organ}}}{m_{\mathrm{TB}}}\right)}_{\text{human}}\right],$$

where *m*_TB_ is the total body mass of the rat or human, and *m*_organ_ is the mass of tissue or organ of the rat or human (see Table 1), and $\frac{\%\mathrm{ID}}{\text{organ}}$ is the percentage of injected dose for the organ in question for the rat or human [7].

**Table 1:** **Organ masses of both rat and human used in the radiodosimetry analysis**[7]

**Organ**	**Organ masses (g)**	
	**Rat**	**Human**
Thyroid	0.287	20
Stomach	5.24	250
Liver	10.59	1,800
Heart	0.98	500
Bladder (contents)	0.89	102
Intestine (ULI)	5.54	220
Intestine (LLI)	3.4	135
Intestine (small)	5	400
Remainder	224	66,573
Total body	251	70,000

The resultant human residence times were then entered into OLINDA/EXM [8], which employs the MIRD method in conjunction with the ICRP publication protocols [8-10]. Specifically, the effective dose (ED) was calculated according to ICRP 60 [9] without the splitting rule, and ICRP 103 [11]. However, there are no published tissue dose factors available for the esophagus, salivary glands, extrathoracic region, lymphatic nodes, oral mucosa, or prostate, which are required for the complete calculation using ICRP 103. Therefore adjustments to the final dose had to be made through increasing the effective dose in proportion to the total tissue weighting factor in order to compensate for the unaccountable organs. Such adjustment, though not ideal, is deemed acceptable according to the code developer of OLINDA/EXM. The percentage increase of effective dose was calculated using the weighting factors in ICRP 103 (see Table 2). The weighting factors for the tissues without dose factors are: esophagus, 0.04; salivary glands, 0.01; additionally, the extrathoracic region, lymphatic nodes, oral mucosa and prostate as a part of remainder, 0.12*(4/13) male and 0.12*(3/13) female. This accounts for a cumulative tissue weighting factor of 0.0869 (or 8.69%) for male, and 0.0777 (or 7.77%) for female, shown by the calculations below.

**Table 2:** **Tissue weighting factors in ICRP 103**[11]

**Tissue/Organ**	**Weighting factor**
Breast, colon, lungs, red marrow, remainder, stomach	0.12
Gonads	0.08
Bladder, liver, esophagus, thyroid	0.04
Bone surfaces, brain, salivary glands, skin	0.01

The following 13 tissues/organs were considered as parts of the ‘Remainder’ in the dosimetry estimate: adrenal glands, extrathoracic region, gall bladder, heart, kidneys, lymphatic nodes, muscle, oral mucosa, pancreas, small intestine, spleen, thymus, and prostate (male), or uterus/cervix (female).

$$\text{Male}:0.04+\frac{4}{13}0.12+0.01=0.0869$$

$$\text{Female}:0.04+\frac{3}{13}0.12+0.01=0.0777$$

### Data analysis and statistics

Average values (mean) and corresponding standard deviation (SD) were calculated with Microsoft Excel (Redmond, WA, USA) and expressed as mean ± SD, and number of measurements is expressed as *n*. The rest of the analysis was performed options using Prism 5 (GraphPad, La Jolla, CA, USA) software. In order to determine the difference among various groups of data, we applied Kruskal-Wallis non-parametric one-way analysis of variance with matched measurement, followed by Dunnett's post-hoc test. When comparing data from this study and literature, a simple one-way analysis of variance was applied, followed by Dunnett's post-hoc test. For comparing imaging and tissue biodistribution measurements, a two-tailed paired Student's *t* test was used. A correlation analysis was also performed on data acquired through the two methods and a Pearson correlation coefficient (*r*) was calculated. A *P* value of <0.05 was considered to be statistically significant.

## Results

### ^123^I-CMICE-013 imaging biodistribution

**Figure 1:**
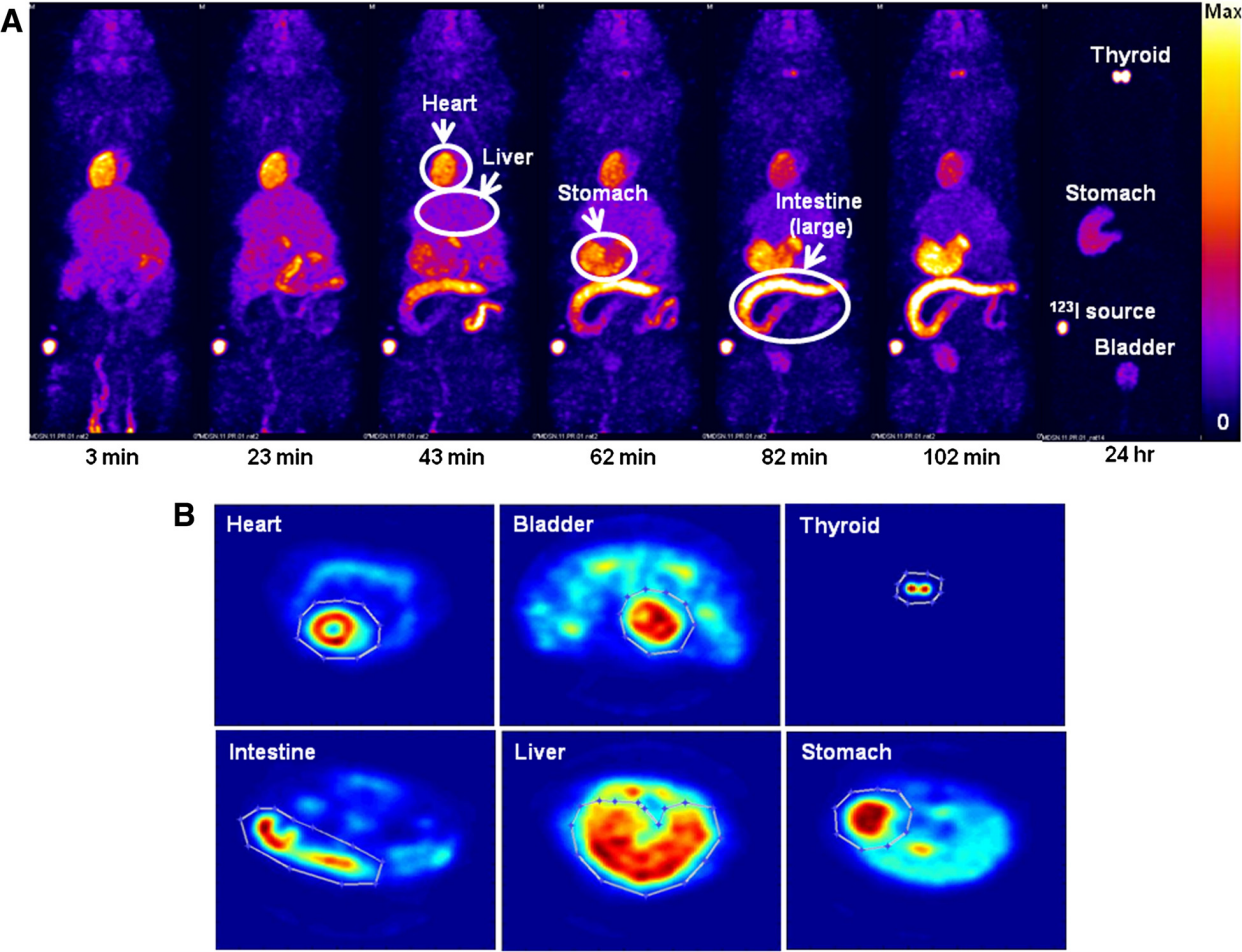
**MicroSPECT images.** A panel of coronal whole-body images of a healthy rat at 3, 23, 43, 62, 82, and 102 min and 24 h after the injection of 45 MBq ^123^I-CMICE-013 **(A)**. The circled areas are indicated as the heart, liver, large intestine, and stomach. The bright spots in the 24-h image were identified as the thyroid, stomach, ^123^I source, and bladder. Volumes of interest (VOIs) for imaging biodistribution analysis were drawn around all areas of focal uptake in the 23- to 102-min summed images **(B)**.

The time course of the distribution of ^123^I-CMICE-013 is shown in the microSPECT images in Figure 1A. Figure 1B illustrates VOIs drawn around the organ of interest for the purpose of quantification of the absorbed activity. Very soon after the injection (3 min), the tracer has started to accumulate in the myocardium, with excellent contrast to the surrounding tissues such as lung and liver. Later on at 43 to 62 min, the radioactivity increases in the stomach and intestine. At 24 h post-injection, most of the activity has been cleared from the heart, intestine and liver.

**Figure 2:**
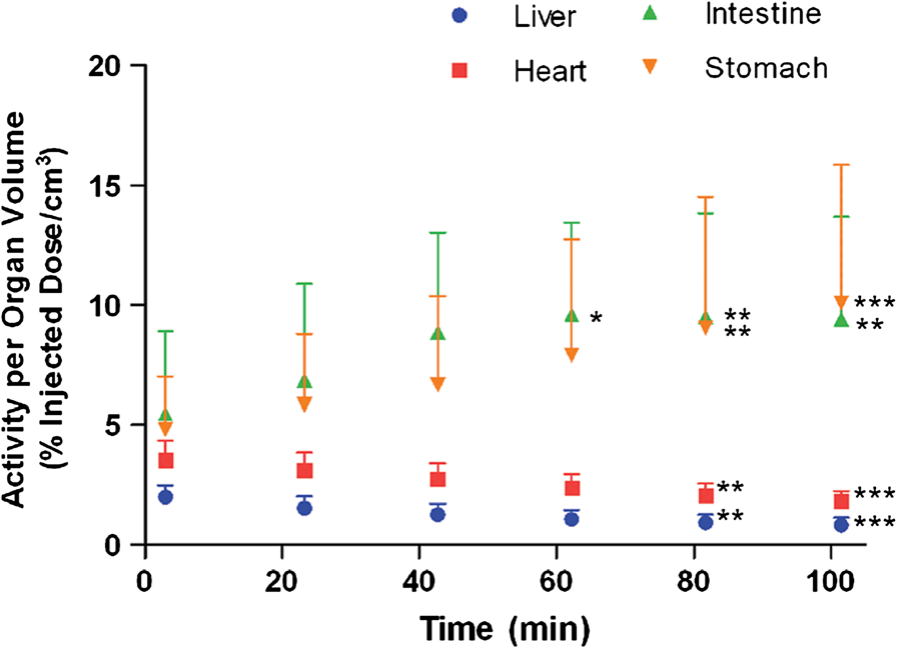
**Representative time-activity density curves.** The percentage of injected dose by organ volume (%ID/cm^3^) from heart, liver, intestine, and stomach, expressed as mean ± SD (*n* = 6), were plotted against time (3 to 102 min) to demonstrate the activity density. * *P* < 0.05, ** *P* < 0.001, *** *P* < 0.0001 versus the data points at 3 min.

The calculated percentage-injected dose values are presented in Table 3. Due to the lack of activity in the lung during the scan, no VOI was drawn at the lung and no %ID/organ value is presented for lung in this table. To better understand the organ activity density, %ID/organ values were divided by the volume of the organ. The volume of organ was estimated as the number of voxels in the VOI surrounding the organ that contained activity greater than the background. Each voxel had a size of 0.6 × 0.6 × 0.6 mm which is equivalent to 2.16E-04 mL or cm^3^. The resultant %ID/cm^3^ plotted against time is presented in Figure 2. The activity density of the myocardium showed a gradual decline that did not become significantly different (*P* < 0.05) until 1 h after the administration of the tracer. The measurements in the intestine and stomach showed an increase over the time and reached 9.423 ± 4.275 %ID/cm^3^ and 10.098 ± 5.763 %ID/cm^3^, respectively, at the end of the scan.

**Table 3:** **Biodistribution of **^**123**^**I-CMICE-013 in healthy Sprague–Dawley rats**

**Organ**	**%ID/organ (mean ± SD)**						
	**2 min**	**23 min**	**43 min**	**62 min**	**82 min**	**102 min**	**24 h**
Thyroid	0.47 ± 0.20	0.48 ± 0.19	0.48 ± 0.17	0.48 ± 0.17	0.49 ± 0.15	0.49 ± 0.14	0.69 ± 0.23
Stomach	6.70 ± 2.25	7.84 ± 1.68	8.71 ± 1.24	10.10 ± 0.98	11.60 ± 1.10	13.09 ± 1.58	1.30 ± 0.34
Liver	17.96 ± 4.46	13.84 ± 4.63	11.39 ± 4.21	9.61 ± 3.59	8.30 ± 3.19	7.39 ± 2.95	n/a
Heart	4.18 ± 0.99	3.69 ± 0.77	3.24 ± 0.70	2.81 ± 0.62	2.43 ± 0.56	2.16 ± 0.48	n/a
Bladder	0.15 ± 0.19	0.42 ± 0.40	0.52 ± 0.49	0.56 ± 0.52	0.61 ± 0.56	0.64 ± 0.57	0.16 ± 0.07
Intestine	4.69 ± 2.78	5.81 ± 2.91	7.63 ± 3.24	8.28 ± 3.18	8.06 ± 3.07	7.91 ± 3.02	0.58 ± 0.33
Sal. gland	2.97 ± 1.12	3.04 ± 1.16	3.06 ± 1.16	3.05 ± 1.16	3.00 ± 1.13	2.95 ± 1.11	n/a

Fourteen animals were divided into two groups. Group no. 1 (*n* = 6) was used in 0- to 2-h imaging and group no. 2 (*n* = 8) was used in 24-h imaging. At 24 h post-injection, no activity was observed in the microSPECT images of the liver, heart, and salivary gland.

**Figure 3:**
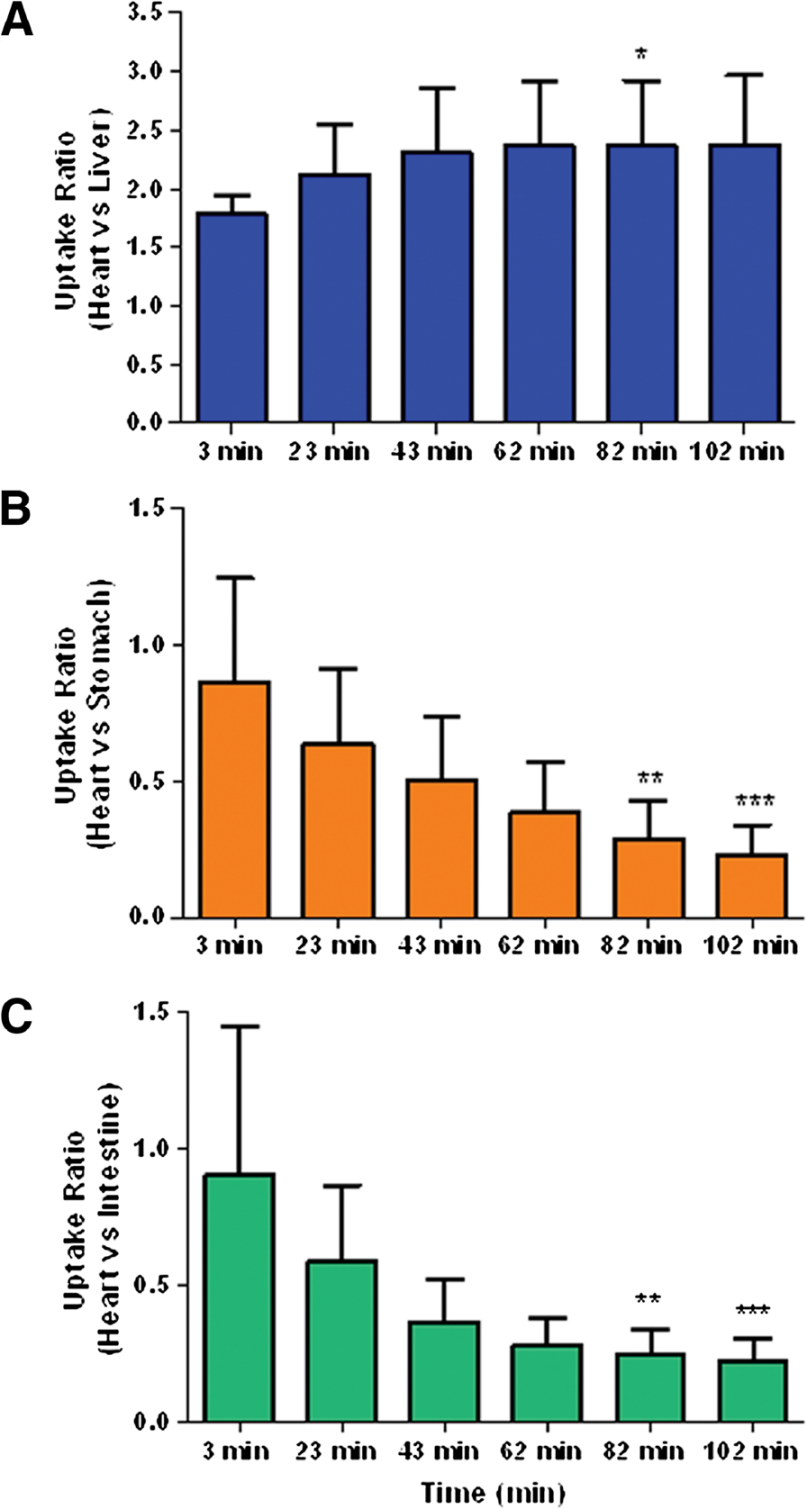
**Heart/organ uptake ratios.** The %ID/cm^3^ of heart was divided by that of liver, stomach, and intestine to calculate the uptake ratio of heart/liver **(A)**, heart/stomach **(B)** and heart/intestine ratios **(C)** at different time points post-injection of the tracer. Data were presented as mean ± SD (*n* = 6). * *P* < 0.05, ** *P* < 0.001, *** *P* < 0.0001 versus the data points at 3 min.

**Figure 4:**
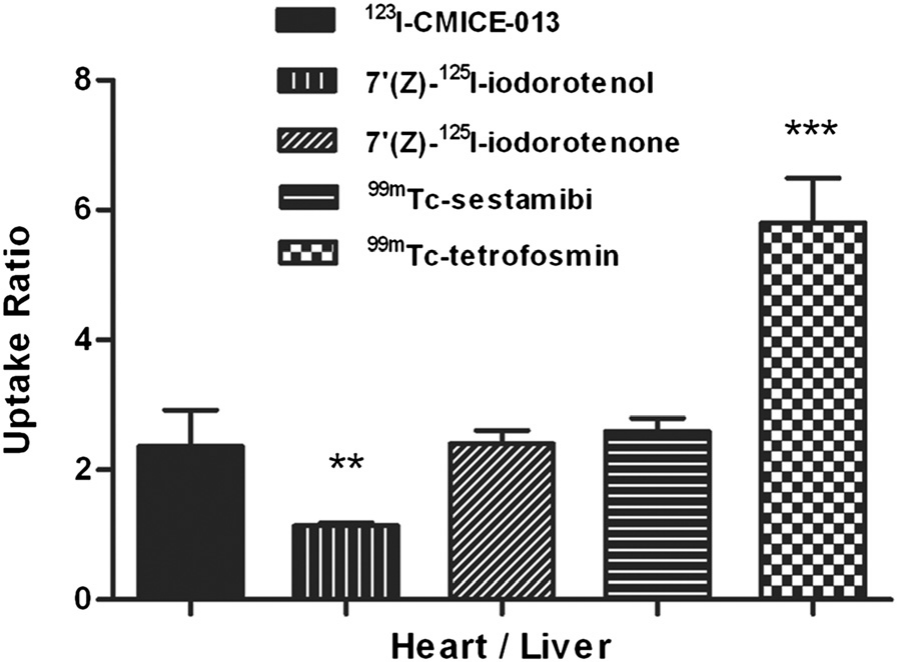
**Heart/liver ratio of **^**123**^**I-CMICE-013 versus other perfusion agents.** The heart/liver ratio of ^123^I-CMICE-013 at 62 min (approximately 1 h) was compared to that of 7′(Z)-^125^I-iodorotenol, 7*'*(Z)-^125^I-iodorotenone, ^99m^Tc-tetrofosmin, and ^99m^Tc-sestamibi [12,13]. The ratio of ^123^I-CMICE-013 was expressed as mean ± SD (*n* = 6); 7*'*(Z)-^125^I-iodorotenol, 7*'*(Z)-^125^I-iodorotenone as mean ± SD (*n* = 3, 7); and ^99m^Tc-tetrofosmin, ^99m^Tc-sestamibi as mean ± SEM (*n* = 5, 5). ** *P* < 0.001, *** *P* < 0.0001 versus ^123^I-CMICE-013.

Uptake ratios were calculated by dividing the activity density of the heart by the other three organs and shown in Figure 3. The heart/liver ratio (Figure 3A) remained between 2 and 2.5 for almost the entire scan and reach a maximum (2.382 ± 0.588) at the last time interval. The heart/stomach ratio (Figure 3B) and heart/intestine (Figure 3C) however demonstrated a decreasing trend - from almost 1 at the beginning to 0.2 at the end (0.223 ± 0.081 and 0.225 ± 0.112, respectively) - indicating the tracer accumulated in both organs. The heart/liver ratio of ^123^I-CMICE-013 (2.365 ± 0.554; Figure 4) appears to be higher or similar to two other rotenone-derived radiotracers 7′(Z)-^125^I-iodorotenol (1.136 ± 0.048) and 7′(Z)-^125^I-iodorotenone (2.402 ± 0.209) from a previous report [12]. It is also close to ^99m^Tc-sestamibi (2.6 ± 0.2) but only around one half of ^99m^Tc-tetrofosmin (5.8 ± 0.7) [13].

**Figure 5:**
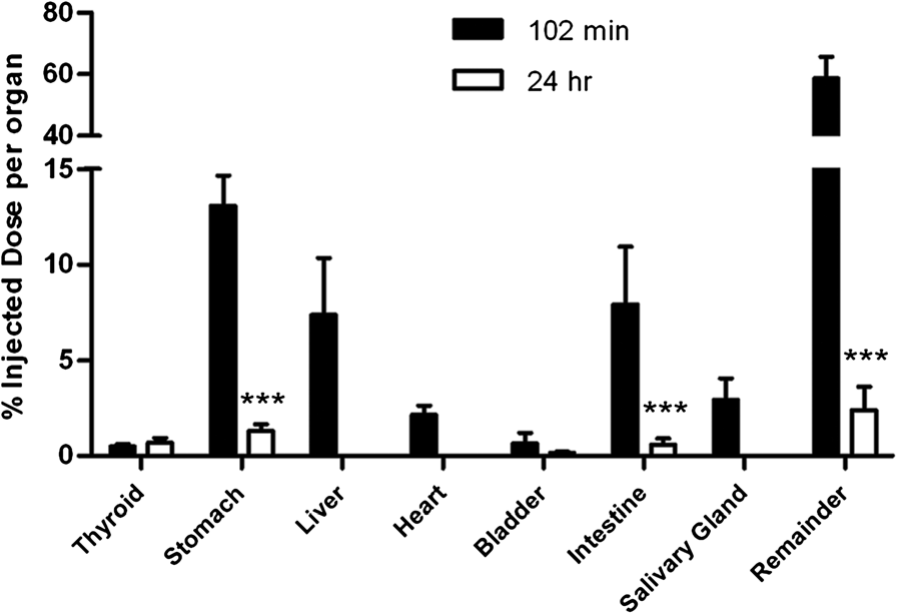
**Tissue uptake of **^**123**^**I-CMICE-013 at 102 min versus 24 h post-injection.** Due to no evident activity in the microSPECT image at 24 h, no %ID/organ values were registered in VOI for the liver, heart, or salivary gland. The %ID/organ values were expressed as mean ± SD. *n* = 6 for 102-min data, *n* = 8 for 24-h data. *** *P* < 0.0001 versus values at 102 min.

A graph of %ID/organ values at 102 min and 24 h showed an overnight clearance of most activity on a whole-body scale (Figure 5). Only four organs (thyroid, stomach, bladder, and intestine) were observed to have activity above background, and among them, both the stomach and intestine showed a significant drop of activity (*P* < 0.0001). The remainder %ID/organ also decreased significantly (*P* < 0.0001) between 102 min and 24 h, indicating a massive excretion of background activity.

### Imaging biodistribution versus tissue biodistribution

**Figure 6:**
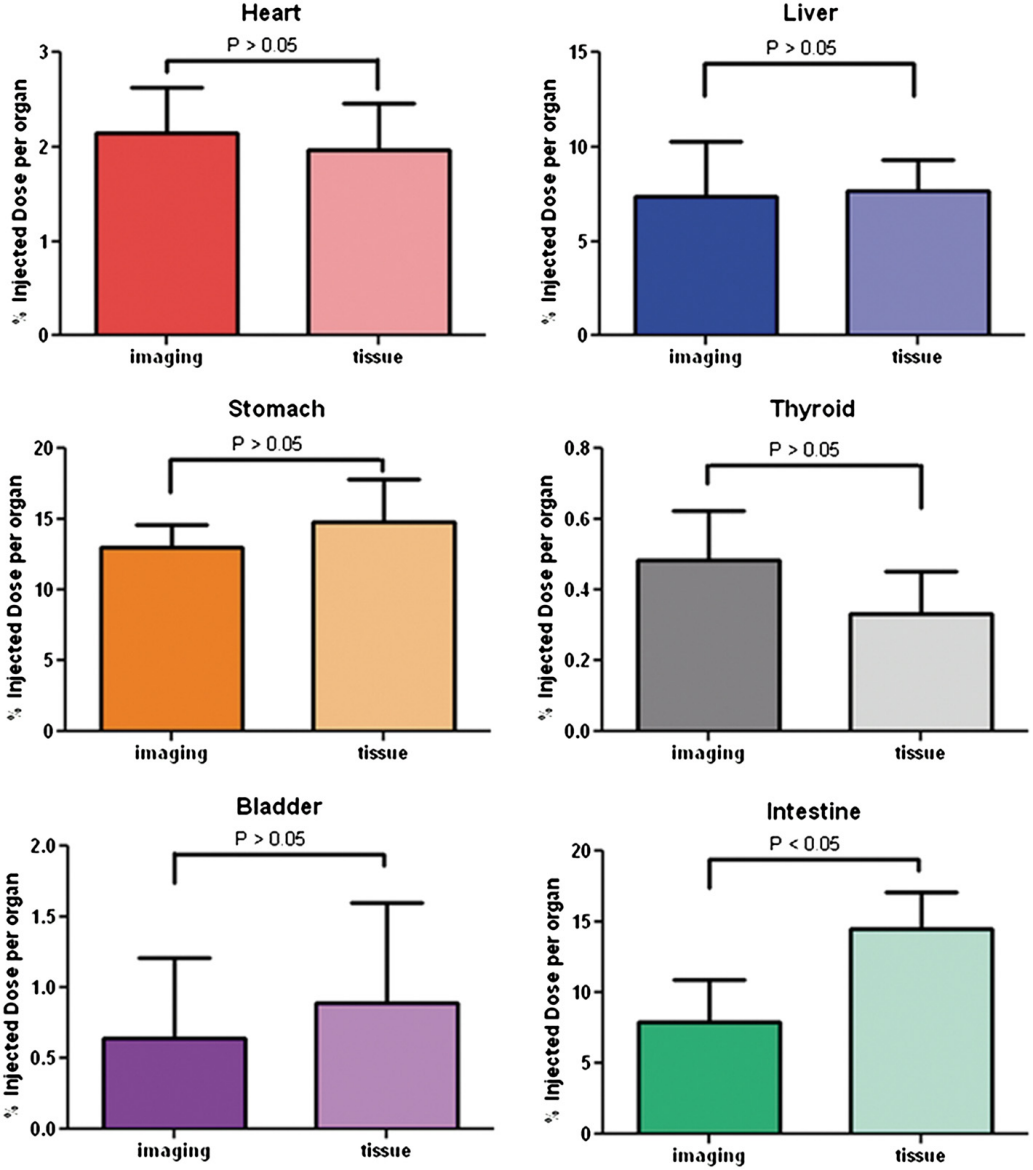
**Comparison of imaging and tissue biodistribution in six organs.** The %ID/organ values were expressed as mean ± SD (*n* = 6). No significant difference was observed except in the intestine.

In order to verify the accuracy of the imaging biodistribution approach, the %ID/organ values calculated from microSPECT images at 2 h post-injection were compared to the %ID/organ values generated from tissue extraction and gamma well counting (Figure 6). Only data from six organs were compared because the salivary gland was not collected for tissue biodistribution. There was no significant difference between the two groups of data for any of the considered organs (*P* > 0.05) except for the intestine (*P* = 0.03).

**Figure 7:**
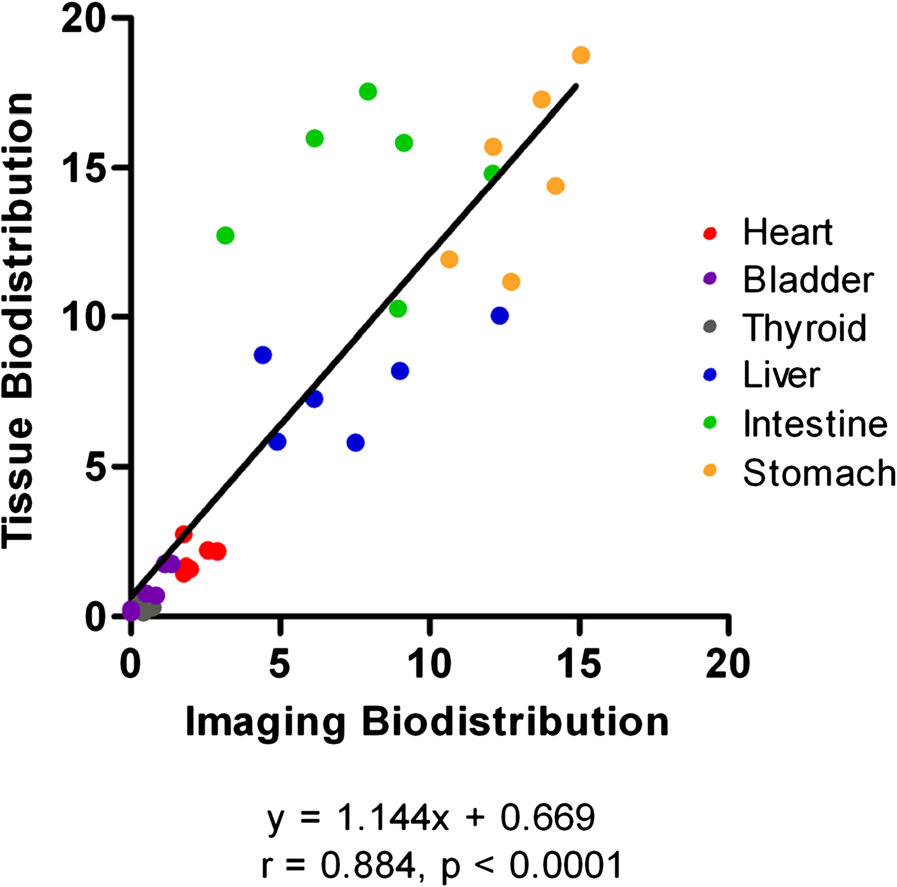
**Correlation between imaging and tissue biodistribution.** Each of the total 36 spots in the graph represents a pair of %ID/organ values per tissue of one of the six animals.

In order to further evaluate the correlation of the data acquired with the two different methods, a Pearson correlation coefficient (*r*) was calculated based on the scatter plots of imaging and tissue biodistribution data. Figure 7 demonstrated a strong correlation overall (*r* = 0.882, *P* < 0.0001). A linear fit to the data has a slope of 1.144 and an intercept of 0.669 %ID/organ.

### Vital sign monitoring

No significant change in respiratory rate or heart rate was observed over the time from 0 to 2 h during the scan. The mean values were 55 ± 13 and 373 ± 34 for the respiratory rate and heart rate, respectively.

### Radiodosimetry analysis

**Figure 8:**
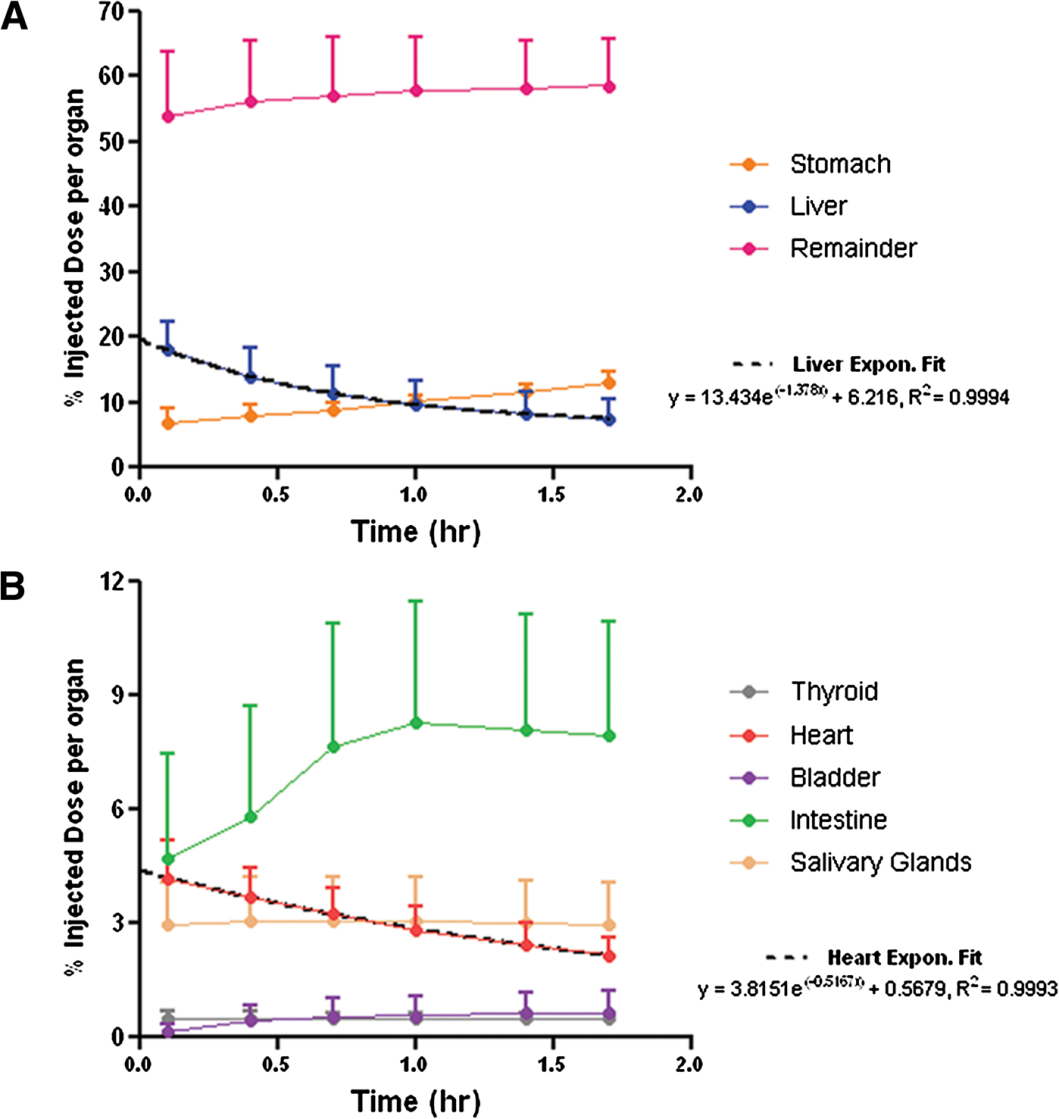
**Time-activity curves of **^**123**^**I-CMICE-013 in seven organs plus remainder.** In order to best demonstrate the percentage of injected dose per organ values of a large scale, the time-activity curves are split into two graphs, **A** and **B**. An exponential fitting curve is fitted for both the liver and the heart. Results were expressed as mean ± SD (*n* = 6).

The averaged %ID/organ values from 3 to 102 min are shown in Figure 8. The residence times (Bq-h/Bq) of organs and remainder are given in Table 4. The heart and liver show clear signs of continuous clearance; therefore, an exponential curve of best fit values was plotted for the two organs and integrated to calculate the residence times. With respect to other organs, given the long physical half life of ^123^I (*t*_1/2_ = 13.2 h), we used the assumption that the remaining activity in the body at 102 min (1.7 h) would decay to zero without any excretion, in order to provide a conservative estimate of the residence times.

**Table 4:** Rat and human residence times

**Organ**	**Residence times (kBq-h/kBq)**	
	**Rat**	**Human**
Thyroid	3.79E-02	9.46E-03
Stomach	9.74E-01	1.66E-01
Liver	2.95E-01	1.79E-01
Heart	9.79E-02	1.80E-01
Bladder (contents)	7.26E-02	2.99E-02
Intestine (ULI)	2.35E-01	3.34E-02
Intestine (LLI)	1.44E-01	2.05E-02
Intestine (small)	2.32E-01	6.07E-02
Remainder	4.79	5.1

Rat residence times were converted to human residence times and OLINDA/EXM was used to calculate the dose to target organs. The value of intestine was split up among the upper large intestine (ULI), lower large intestine (LLI), and the small intestine (SI), by proportion to the mass of these three organs. The salivary glands were added to the remainder in this analysis.

Table 5 listed analysis results for the whole-body effective dose (6.99E-03 mSv/MBq for ICRP 60; 7.15E-03 mSv/MBq for ICRP 103) as well as the doses to various target organs. The stomach was seen to accumulate the highest dose 1.36E-02 mSv/MBq for male and 1.64E-02 mSv/MBq for female, seconded by the thyroid 1.24E-02 mSv/MBq for male and 1.44E-02 mSv/MBq for female. The dose of the intestine was represented by ULI 6.92E-03 mSv/MBq for male and 8.44E-03 mSv/MBq for female, LLI 6.63E-03 mSv/MBq for male and 7.96E-03 mSv/MBq for female, and SI 6.82E-03 mSv/MBq for male and 7.80E-03 mSv/MBq for female. Considering the two major metabolism organs, the dose of kidney was 4.87E-03 mSv/MBq for male and 6.08E-03 mSv/MBq for female, whereas liver was 5.76E-03 mSv/MBq for male and 7.48E-03 mSv/MBq for female. Based on this estimate and the available data from ICRP 80 [5] (Table 6), ^123^I-CMICE-013 has a modest radiodosimetric profile.

**Table 5:** **Estimated effective doses of **^**123**^**I-CMICE-013 for male and female humans**

**Organ**	**Male**		**Female**	
	**Equivalent dose to target organ (mSv/MBq)**	**Weighted equivalent dose (mSv/MBq)**	**Equivalent dose to target organ (mSv/MBq)**	**Weighted equivalent dose (mSv/MBq)**
Adrenals	5.35E-03	2.68E-05	6.72E-03	3.36E-05
Brain	3.84E-03	1.92E-05	4.86E-03	2.43E-05
Breasts	3.40E-03	1.70E-04	4.36E-03	2.18E-04
LLI	6.63E-03	7.96E-04	7.96E-03	9.55E-04
SI	6.82E-03	3.41E-05	7.80E-03	3.90E-05
Stomach	1.36E-02	1.63E-03	1.64E-02	1.97E-03
ULI	6.92E-03	3.46E-05	8.44E-03	4.22E-05
Kidneys	4.87E-03	2.44E-05	6.08E-03	3.04E-05
Liver	5.76E-03	2.88E-04	7.48E-03	3.74E-04
Lungs	4.81E-03	5.77E-04	6.33E-03	7.60E-04
Muscle	4.17E-03	2.09E-05	5.16E-03	2.58E-05
Pancreas	6.49E-03	3.25E-05	8.03E-03	4.02E-05
Red marrow	3.91E-03	4.69E-04	4.73E-03	5.68E-04
Osteogenic cells	1.37E-02	1.37E-04	1.76E-02	1.76E-04
Skin	2.89E-03	2.89E-05	3.53E-03	3.53E-05
Spleen	5.29E-03	2.65E-05	6.58E-03	3.29E-05
Testes (m)/Ovary (f)	3.92E-03	7.84E-04	6.77E-03	1.35E-03
Thymus	4.92E-03	2.46E-05	6.19E-03	3.10E-05
Thyroid	1.24E-02	6.20E-04	1.44E-02	7.20E-04
Bladder	6.56E-03	3.28E-04	8.28E-03	4.14E-04
Uterus	5.58E-03	2.79E-05	6.63E-03	3.32E-05
ED ICRP 60	6.99E-03			
ED ICRP 103	7.15E-03			

**Table 6:** **Effective doses of **^**123**^**I-based tracers listed in ICRP 80**[5]

**Tracer**	**EDE (mSv/MBq)**
Iodide (thyroid blocked, uptake 0%)	1.10E-02
Iodide (thyroid uptake 35%)	2.20E-01
Iodoamphetamine (IMP)	2.70E-02
Iodine-labeled fibrinogen	2.00E-02
Iodine-labeled albumin (HSA)	2.00E-02
Iodine-labeled microaggregated albumin (MIAA)	1.80E-02
Hippuran	1.20E-02
Hippuran, bladder emptied 1 h after administration	4.60E-03
Hippuran, bladder emptied 0.5 h after administration	5.90E-03
Metaiodobenzylguanidine (MIBG)	1.30E-02
Sodium rose bengal	5.90E-02

It should be noted that our assessment of human equivalent effective dose is based on the converted residence times from rat data, which assumes that the tracer biokinetics will be similar between small rodents and humans. Although this approach can be utilized to evaluate the dosimetry of novel radiotracers in preclinical studies [14,15], the biokinetics of tracers are often different between rodents and humans. Thus, the dosimetry derived from rat studies is, at best, a rough estimate. Future dosimetry investigations in other species including humans are required to confirm the safety of ^123^I-CMICE-013.

## Discussion

^123^I-CMICE-013 represents a novel radioiodine-labeled rotenone-derived MPI agent and is currently under the investigation in our laboratory. In this study, by performing quantitative microSPECT image analysis, we showed high heart uptake of ^123^I-CMICE-013 and a favorable biodistribution profile. Images showed a rapid myocardial uptake soon after administration, with little or no interference from surrounding tissues such as the liver and lung. The heart/liver uptake ratio was similar to other rotenone-based MPI tracers 7′(Z)-^125^I-iodorotenol and 7′(Z)-^125^I-iodorotenone [12], as well as the benchmark agent ^99m^Tc-sestamibi [13]. Heart/stomach and heart/intestine ratios remained moderate within the first 1 h post-injection of the tracer, which allow for a good imaging window. At 24 h post-injection, a good clearance of tracer on the whole-body scale was also observed.

**Figure 9:**
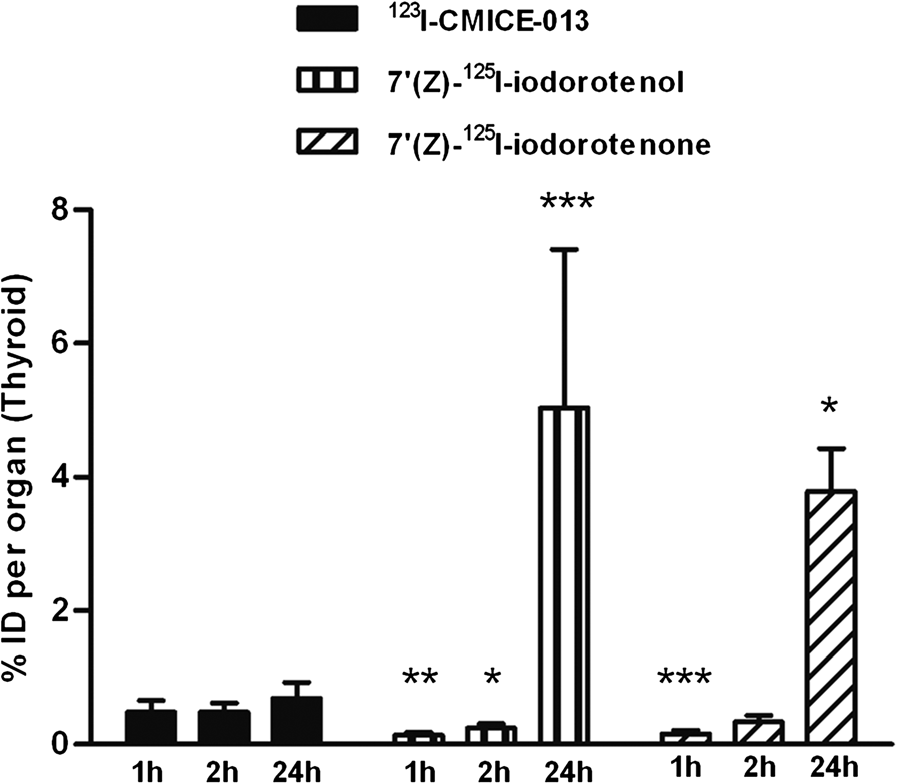
**Thyroid uptake of **^**123**^**I-CMICE-013 versus two other perfusion agents.** The thyroid uptake values (%ID/organ) of two 7*'*(Z)-^125^I-iodorotenol and 7′(Z)-^125^I-iodorotenone were compared to ^123^I-CMICE-013 at 1, 2, and 24 h post-injection. A 102-min data of ^123^I-CMICE-013 was used as 2-h data in this graph. All data were expressed as mean ± SD. The number of animals at each time point (in the order of 1, 2, 24 h) is ^123^I-CMICE-013 *n* = 6, 6, 8; 7*'*(Z)-^125^I-iodorotenol *n* = 3, 4, 4; 7*'*(Z)-^125^I-iodorotenone *n* = 7, 6, 4. * *P* < 0.05, ** *P* < 0.001, *** *P* < 0.0001 versus ^123^I-CMICE-013 at the same time point.

Depending on the chemical nature, dehalogenation *in vivo* of radioiodinated tracers occurs at different paces, which eventually leads to the various degrees of thyroid accumulation of radioactive iodine. We compared the thyroid uptake value of ^123^I-CMICE-013 to the data of two closely related compounds 7′(Z)-^125^I-iodorotenol and 7′(Z)-^125^I-iodorotenone [12], which were acquired with tissue biodistribution method (Figure 9). At 1 h, ^123^I-CMICE-013 (0.483 ± 0.173 %ID/organ) activity was about 3 times that of 7′(Z)-^125^I-iodorotenol (0.139 ± 0.047 %ID/organ) and 7′(Z)-^125^I-iodorotenone (0.154 ± 0.057 %ID/organ); whereas at 24 h, ^123^I-CMICE-013 (0.693 ± 0.235 %ID/organ) was only one seventh of 7′(Z)-^125^I-iodorotenol (5.037 ± 0.341 %ID/organ) and one sixth of 7′(Z)-^125^I-iodorotenone (3.784 ± 0.647 %ID/organ). Such difference in thyroid uptake may indicate a slow and steady pace of tracer degradation for ^123^I-CMICE-013, albeit the different evaluation methods (imaging versus tissue) would have also contributed to the discrepancy in the results between the two studies.

We also compared the radiation dosimetry of ^123^I-CMICE-013 to 7′(Z)-^123^I-iodorotenone, an ^123^I-labeled rotenone derivative. Broaisat et al. performed a preliminary radiation dose estimate on dogs injected with 7′(Z)-^123^I-iodorotenone using MIRDOSE 3.1 [16]. Selected organs were evaluated, including the gallbladder wall (3.08E-01 mSv/MBq), stomach (1.24E-01 mSv/MBq), heart wall (8.11E-03 mSv/MBq), kidneys (1.08E-02 mSv/MBq), liver (1.08E-02 mSv/MBq), and salivary gland (1.78E-01 mSv/MBq). Compared to the matched organs in our study, the dose values of ^123^I-CMICE-013 injection were overall lower: stomach 1.36E-02 mSv/MBq for male; 1.64E-02 mSv/MBq for female, kidneys 4.87E-03 mSv/MBq for male and 6.08E-03 mSv/MBq for female, and liver 5.76E-03 mSv/MBq for male and 7.48E-03 mSv/MBq for female. As for all radioiodine-based tracers, the radiation dosimetry in the thyroid always attracts attention due to the potential for tracer de-iodination and subsequent uptake in the thyroid. Our estimate suggested that ^123^I-CMICE-013 created a mild dose to thyroid (1.24E-02 mSv/MBq for male; 1.44E-02 mSv/MBq for female), only about one-fifth of that of 7′(Z)-^123^I-iodorotenone (7.03E-02 mSv/MBq). Nevertheless, as is frequently done with ^123^I-compound in current clinical use, a thyroid-blocking agent could be administered to further reduce thyroid exposure.

Our radiodosimetry analysis also indicated that ^123^I-CMICE-013 created a moderate whole-body effective dose that is lower than both benchmark MPI tracers ^99m^Tc-sestamibi (9.0E-03 mSv/MBq at rest) and ^99m^Tc-tetrofosmin (7.6E-03 mSv/MBq at rest) [5]. It is also well within the range of ICRP 80 listed ^123^I-based tracers [5] and highly competitive among other novel tracers that are currently under investigation. ^123^I-MNI-420, a SPECT tracer for imaging A2A receptor in brain, had an effective dose of 3.60E-02 mSv/MBq [17]. The effective dose of ^123^I-βCIT (dopamine transporter imaging agent) and ^123^I-ADAM (serotonin transporter imaging agent) were estimated to be 3.10E-02 mSv/MBq [18] and 3.37E-02 mSv/MBq [19]. A dosimetry analysis based on a canine model injected with 7′(Z)-^123^I-iodorotenone revealed that the tracer gave an effective dose of 2.97E-03 mSv/MBq [16]. Given that the current estimate for^123^I-CMICE-013 was based on a limited number of healthy rats, data should be interpreted as preliminary and future studies in large animals, and eventually, human subjects are critical for a more precise and accurate measure of radiodosimetry.

This study describes the characterization of the biodistribution profile of ^123^I-CMICE-013 using microSPECT image-based quantification. For studying radiotracer uptake in small animal models, the quantitative SPECT imaging has several advantages over purely tissue-sampling-based methods. As a non-invasive method, it allows repeat three-dimensional imaging of the same animal over multiple time points [20,21]. We acquired six time points after a single injection of tracer for each animal. This reduces the amount of tracer product that needs to be formulated for evaluation, decreases the number of animals needed to obtain the data points, and improves the consistency in the time-activity curves by removing any subject-to-subject variability. The images provide a full *in vivo* picture of the entire animal at each time point, which removes any distortion of distribution or losses of tracer that may be caused by sacrificing the animal and tissue sectioning. Finally the image ensures that samples can be taken from all relevant sites of focal uptake, reducing the possibility of not acquiring samples from sites of unexpected tracer accumulation. For instance, the salivary glands were not collected during tissue extraction in this study because it was not realized a priori that ^123^I-CMICE-013 would accumulate in these tissues.

The data from image analysis is largely consistent with those from tissue sampling. For most organs, the %ID/organ values showed no significant difference between the data from two methods. The correlation analysis also showed an excellent correlation (*r* = 0.882) overall. The one exception was the uptake in the intestine. A limitation of the quantitative SPECT imaging approach is a reduced sensitivity [22]. As the organs are evaluated *in situ*, the presence of uptake in surrounding or nearby tissues generates a background that reduces sensitivity compared to that obtained when the tissue is removed from the subject. We suspect that the differences seen in the intestine were due to diffuse uptake in the intestinal walls that was not resolved over background in the images and thus not included in the organ activity estimate. An additional contributing factor could be the limited resolution of the camera [22]. Partial volume effects will dilute the apparent uptake in small structures and could make small organs with low levels of uptake difficult to distinguish from background [23]. A final limitation is the difficulty of identifying anatomical structures and boundaries of structures from the tracer distribution image. We mitigated this using a co-registered CT scan for anatomical localization, which has been proven to greatly improve the ease of identification of anatomical regions [24-26]. Due to the fact that CT still suffers from poor soft-tissue contrast, ultimately the use of a co-registered MRI might prove more beneficial. Without a definite measure of tissue boundaries, estimates of organ mass are also reduced in accuracy and this increases the uncertainty of converting data into measures of %ID/g of tissue. Thus, in lieu of a purely image-based or tissue sample-based approach, a hybrid method such as that used in this study may provide the advantages of both. Using multiple image points and tissue sampling coincident with the final time provides the opportunity to specifically target sampling of small organs and also provides accurate measures of organ volumes and weights. The good consistency and correlation between the data from the two methods suggested that our hybrid imaging-based quantification technique is a powerful tool to monitor the kinetics distribution of SPECT radiotracers.

## Conclusions

This study represents an exploratory investigation of the ^123^I-CMICE-013 biodistribution profile using *in vivo* whole-body imaging and validation with tissue analysis. Our tracer under development demonstrated biodistribution features of a desirable myocardial perfusion imaging agent, such as the high myocardium uptake, rapid liver clearance, and lack of lung activity. Furthermore, results from radiodosimetric analysis suggest low organ doses and a modest whole-body dose, which provides a favorable justification for prospective dosimetry studies in larger animals and humans.

## Competing interests

Yin Duan, Lihui Wei, Corinne Bensimon, and Pasan Fernando were employees of Nordion Inc. during this study.

## Authors’ contributions

LW and DD contributed to the synthesis of the tracer. JL, LW, and DD were responsible for the imaging study and *ex vivo* tissue assays. RGW and KS wrote and tested the image quantification program and performed image analyses. CH performed radiation dosage estimation base on the imaging data. PF and RGW contributed to statistical analysis of biodistribution data. CB and TDR supervised and coordinated the study and manuscript preparation. YD drafted the manuscript; CH, JL, LW, RGW, and TDR participated in the writing. All authors reviewed and approved the final manuscript.
